# A Pan-Cancer *In Silico* Analysis of the COVID-19 Internalization Protease: Transmembrane Proteaseserine-2

**DOI:** 10.3389/fgene.2022.805880

**Published:** 2022-02-25

**Authors:** Yiming Meng, Jing Sun, Guirong Zhang, Tao Yu, Haozhe Piao

**Affiliations:** ^1^ Department of Central Laboratory, Liaoning Cancer Hospital and Institute, Cancer Hospital of China Medical University, Shenyang, China; ^2^ Department of Biobank, Liaoning Cancer Hospital and Institute, Cancer Hospital of China Medical University, Shenyang, China; ^3^ Department of Medical Imaging, Liaoning Cancer Hospital and Institute, Cancer Hospital of China Medical University, Shenyang, China; ^4^ Department of Neurosurgery, Liaoning Cancer Hospital and Institute, Cancer Hospital of China Medical University, Shenyang, China

**Keywords:** COVID-19, tmprss2, expression, methylation, correlation

## Abstract

The new coronavirus (2019-nCoV) is an emerging pathogen that can cause severe respiratory infections in humans. It is worth noting that many of the affected COVID-19 patients have malignant tumors. In addition, cancer has been identified as a personal risk factor for COVID-19. Transmembrane proteaseserine-2 (TMPRSS2) is a crucial host protease that mediates S protein activation and initially promotes virus entry into host cells. Moreover, it is abnormally expressed in a variety of tumors. However, the systematic analysis of TMPRSS2 aberrations in human cancer remains to be elucidated. Here, we analyzed the genetic changes, RNA expression, and DNA methylation of TMPRSS2 in more than 30 tumors. It has been reported that TMPRSS2 is overexpressed in tumors such as prostate adenocarcinoma (PRAD), and in contrast, the expression of TMPRSS2 is decreased in tumors such as head and neck cancer (HNSC). In addition, TMPRSS2 low DNA methylation was also found in most of these TMPRSS2 high-expressing tumors in this study. Clinical studies have found that there is a significant correlation between the expression of TMPRSS2 and the prognosis of some tumor patients. The expression of TMPRSS2 is also related to the infiltration of cancer-related fibroblasts, and the potential pathways and functional mechanisms were analyzed through KEGG/GO enrichment. In the end, our study planned the genetic and epigenetic variation of TMPRSS2 in human malignant tumors for the first time and provided a relatively comprehensive understanding of the carcinogenic effects of TMPRSS2.

## Introduction

The pandemic respiratory disease sweeping through 2020 and 2021 is a new type of coronavirus pneumonia (coronavirus disease 2019, COVID-19) caused by severe acute respiratory syndrome coronavirus type 2 (SARS-CoV-2) (Surkova et al., 2020). Current studies have proven that the invasion of SARS-CoV-2 into host cells mainly depends on the activation of viral spike protein (S protein) by certain proteases (Ward et al., 2020). Transmembrane proteaseserine-2 (TMPRSS2) is a key host protease that mediates S protein activation and initially promotes virus entry into host cells (Hoffmann et al., 2020). Moreover, it is abnormally expressed in a variety of tumors. Hence, cancer has been identified as an individual risk factor for COVID-19 (Stopsack et al., 2020). In 1997, Antonarakis et al. identified the TMPRSS2 gene for the first time and found that the gene encodes a multimeric protein with a serine protease domain (Paoloni-Giacobino et al., 1997). Since then, due to the high expression of the TMPRSS2 gene in the prostate, research on TMPRSS2 has mainly focused on the related diseases of prostate cancer (Hong et al., 2020). Due to the impact of the new coronavirus epidemic, coupled with the expression of TMPRSS2 in the epithelial cells of the respiratory system, enthusiasm for research on TMPRSS2 has further increased (Kimura et al., 2020). However, the systematic analysis of TMPRSS2 aberrations has not been characterized in human cancers. Then, we planned a pan-cancer analysis of TMPRSS2 in malignant tumors.

Our study is the first to explore the TCGA database to perform pan-cancer analysis on TMPRSS2. We also included a set of factors, such as gene expression, survival status, DNA methylation, genetic changes, protein phosphorylation, immune infiltration, and related cell pathways, to study the potential molecular mechanisms of TMPRSS2 in the pathogenesis or clinical prognosis of different cancers.

## Materials and Methods

### Analysis of Genetic Changes

This study obtained the pan-cancer data of “TMPRSS2” from the cBioPortal database (https://www.cbioportal.org/) (Gao et al., 2013). In the “Cancer Type Summary” module, we observed the change frequency, mutation type, and CNA (copy number change) results of all TCGA tumors. The mutation site information can be displayed in the protein structure diagram or 3D (three-dimensional) structure through the “Mutations” module. We also took advantage of the “comparison” module to obtain data on overall survival and disease-free survival in TCGA cancer cases with or without TMPRSS2 gene alterations.

### Survival Prognosis Analysis

The GEPIA2 database (http://gepia.cancer-pku.cn/index.html), also known as the gene expression interactive analysis database, was researched and developed by the team of Professor Zemin Zhang from Peking University (Tang et al., 2017). Gene expression analysis was based on tumor and normal samples from the TCGA database. We utilized the “survival map” module of GEPIA2 to obtain the differential expression of TMPRSS2 in tumors and corresponding normal tissues, survival differences, and other related genes. OS (overall survival) and DFS (disease-free survival) saliency map data in all TCGA tumors.

First, the GEPIA2 database further verified the TMPRSS2 expression results and obtained the available experimentally determined TMPRSS2 binding protein. We operated the “Similar Gene Detection” module of GEPIA2 to obtain the top 100 targeted genes related to TMPRSS2 based on all TCGA tumor and normal tissue datasets. We also applied the “Correlation Analysis” module of GEPIA2 to perform paired gene Pearson correlation analysis on TMPRSS2 and selected genes. The *p* value and correlation coefficient (R) are exhibited in the plots. In addition, we used the “Gene_Corr” module of TIMER2 to provide heatmap data for selected genes, including the partial correlation (cor) and *p* value in the purity-adjusted Spearman rank correlation test. In addition, we combined the two sets of data for KEGG pathway analysis and GO enrichment analysis.

### Methylation Level Analysis

The UALCAN portal (http://ualcan.path.uab.edu/analysis-prot.html) (Chandrashekar et al., 2017) is an effective tool for online analysis and mining cancer data and is mainly used for the Cancer Genome Atlas (TCGA) project. This website can be regarded as a platform for computer verification of target genes and identification of tumor subgroup-specific candidate biomarkers. The methylation level between the primary tumor and normal tissues was entered by “TMPRSS2.” The available datasets for six tumors were selected, namely, breast cancer, ovarian cancer, colon cancer, clear cell renal cell carcinoma, uterine corpus endometrial carcinoma, and lung adenocarcinoma.

### Gene Expression Analysis

TMPRSS2 was searched in the “Gene_DE” module by running the GRPIA2 database to observe the difference in TMPRSS2 expression between the tumor and adjacent normal tissues. Different tumors or specific tumor subtypes of the TCGA project were observed. Through the “Pathological Staging Diagram” module, a violin diagram of the expression of all TCGA tumors in different pathological stages (stage I, stage II, stage III, and stage IV) was obtained.

### Immune Infiltration Analysis

TIMER2.0 is a database (http://timer.cistrome.org/) that can comprehensively analyze tumor and immune interactions (Li et al., 2017). The database covers 32 types of tumors and provides online analysis, including gene expression, clinical results, somatic mutations, and somatic copy number changes. The relationship between TMPRSS2 expression and immune infiltration in all TCGA tumors was explored through the “immune gene” module. TIMER, CIBERSORT, CIBERSORT-ABS, QUANTISEQ, XCELL, MCPCOUNTER, and EPIC algorithms were applied to the estimation of immune infiltration. The *p* value and the partial correlation (cor) value were obtained through the Spearman rank correlation test with purity adjustment.

### Analysis of the TMPRSS2 Interaction Protein Network and the Related Biological Processes

The STRING database (https://www.string-db.org/) (von Mering et al., 2003) is a well-known database for predicting protein–protein interactions. The database mainly predicts protein interactions through computational prediction, information transfer between different species, and aggregation of other database information. The STRING database was used to analyze the protein network interacting with TMPRSS2 and the biological processes involved in these interacting proteins.

The Interactive Venn diagram viewer (Bardou et al., 2014) and Database for Annotation, Visualization and Integrated Discovery (DAVID) (https://david.ncifcrf.gov/) online analysis tool (Jiao et al., 2012) were utilized for gene ontology (GO) analysis and Kyoto Encyclopedia of Gene and Genome (KEGG) pathway enrichment. Among them, GO functional enrichment analysis included cell composition, biological process, and molecular function enrichment analysis of genes, thereby obtaining a significantly enriched signaling pathway (*p* < 0.05). The smaller the *p* value, the higher the significance.

## Results

### The Expression Level and Genetic Variation of TMPRSS2 in Each Tumor

**FIGURE 1:**
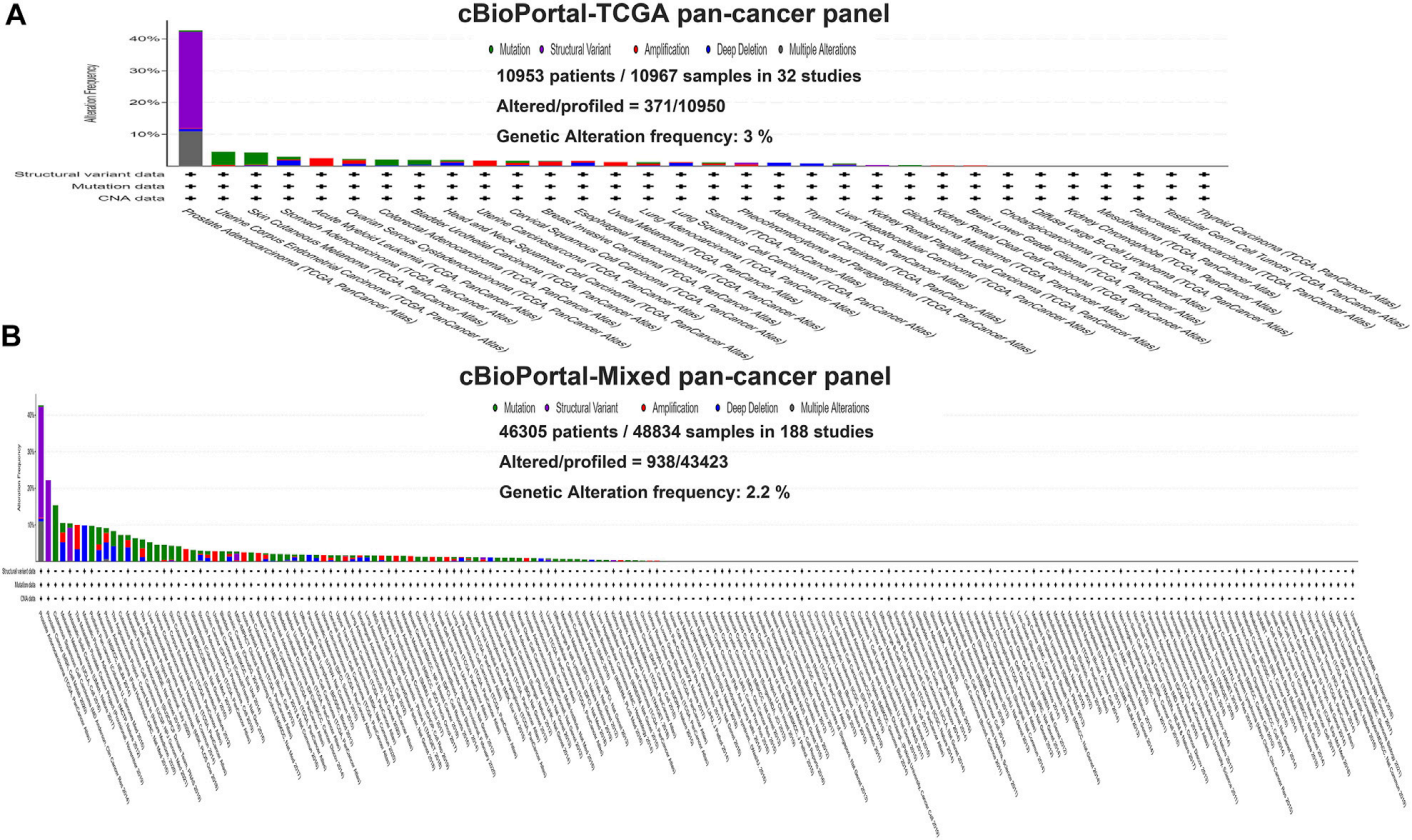
Genetic variation of TMPRSS2 in tumors. **(A)** The TCGA pan-cancer panel was obtained from the cBioPortal dataset, which included 10,953 patients/10967 samples in 32 studies. **(B)** The mixed pan-cancer panel was obtained from the cBioPortal dataset, which also included 46,305 patients/48,834 samples in 188 studies.

**FIGURE 2:**
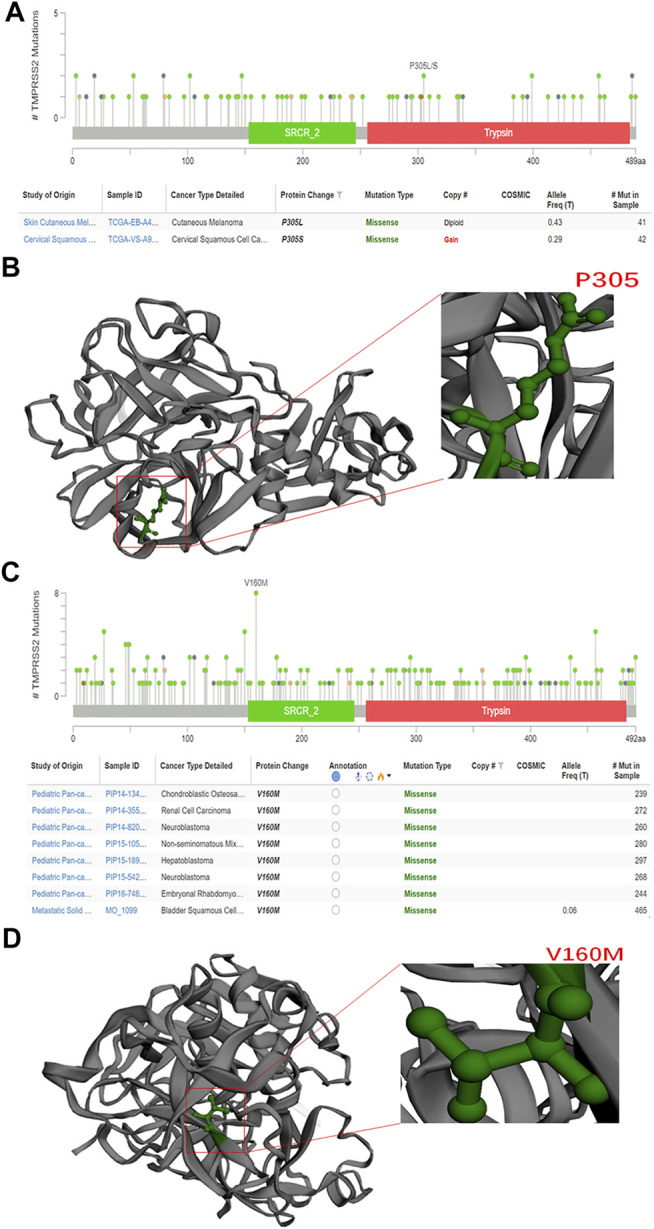
Methylation level of TMPRSS2 in pan-cancer. **(A)** TMPRSS2 promoter DNA methylation probe. **(B)** Three tumors with high expression of TMPRSS2 showed reduced levels of TMPRSS2 DNA methylation, including colon adenocarcinoma (COAD), prostate adenocarcinoma (PRAD), and rectal adenocarcinoma (READ), **p < 0.01. **(C)** Tumors with downregulated TMPRSS2 expression, including breast invasive carcinoma (BRCA), esophageal carcinoma (ESCA), head and neck squamous cell carcinoma (HNSC), kidney renal clear cell carcinoma (KIRC), kidney renal papillary cell carcinoma (KIRP), lung squamous cell carcinoma (LUSC), sarcoma (SARC), skin cutaneous melanoma (SKCM), and thyroid carcinoma (THCA), exhibit increased levels of DNA methylation. **p < 0.01.

**FIGURE 3:**
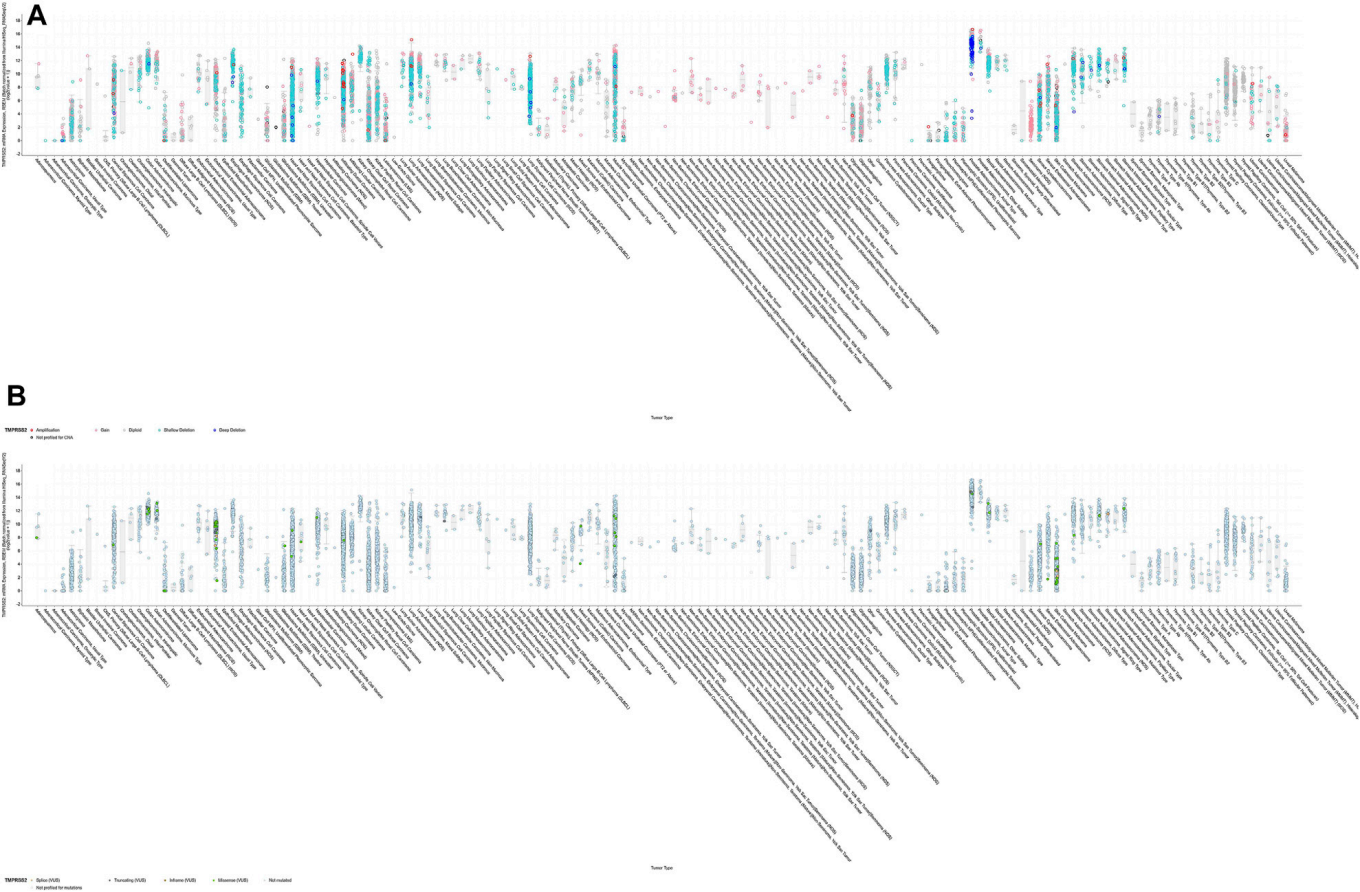
RNA expression of TMPRSS2. **(A)** Exploration of the differential expression of TMPRSS2 through the GEPIA2 database (upper panel; red, tumor samples; green, normal samples) and the UALCAN database (lower panel; blue, tumor samples; red, normal samples). **(B)** TMPRSS2 is overexpressed in colon adenocarcinoma (COAD), cervical squamous cell carcinoma and endocervical adenocarcinoma (CESC), kidney chromophobe (KICH), prostate adenocarcinoma (PRAD), uterine corpus endometrial carcinoma (UCEC), uterine carcinosarcoma (UCS), and rectum adenocarcinoma (READ). **(C)** TMPRSS2 is downregulated in breast invasive carcinoma (BRCA), esophageal carcinoma (ESCA), head and neck squamous cell carcinoma (HNSC), kidney renal clear cell carcinoma (KIRC), kidney renal papillary cell carcinoma (KIRP), lung squamous cell carcinoma (LUSC), sarcoma (SARC), skin cutaneous melanoma (SKCM), testicular germ cell tumors (TGCTs), and thyroid carcinoma (THCA).

In this study, we exploited the cBioPortal database to study the mutations of TMPRSS2 in different cancers, including the mutation site, type, amino acid changes, and the corresponding three-dimensional structure of the protein. To study the relationship between TMPRSS2 copy number changes in different cancers and gene expression, we looked for genes that were coexpressed with TMPRSS2 and presented them in the form of a scatter plot sequentially. The survival curve of TMPRSS2 was searched to lay the foundation for the analysis of survival prognosis. The results were obtained from the cBioPortal dataset, including 10,953 patients/10967 samples in more than 30 studies (Figure 1). According to the TCGA and mixed pan-cancer databases, the main mutation sites of TMPRSS2 are P305 L/S and V160 M (Figure 2), and the mutation of TMPRSS2 in different tumors was also analyzed and is shown in Figure 3.

### Differential Expression and Survival Analysis of TMPRSS2

**FIGURE 4:**
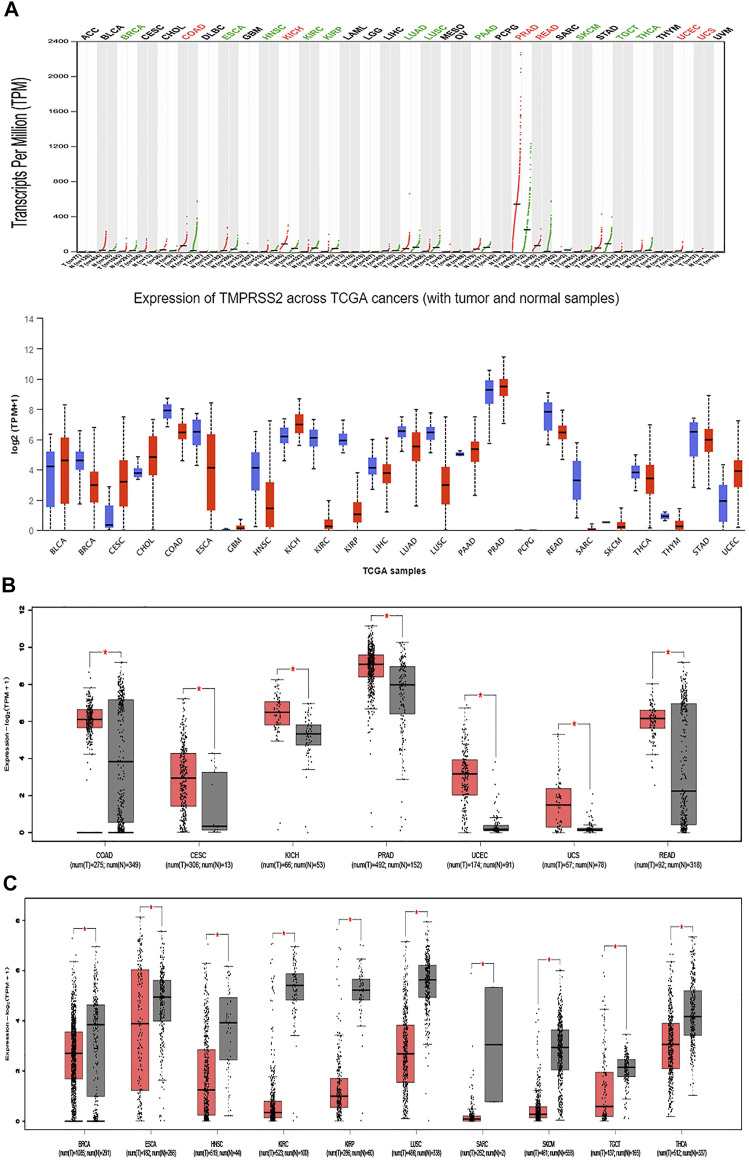
Overall survival (OS) data for malignant tumors overexpressing TMPRSS2. **(A)** Survival picture of tumors overexpressing TMPRSS2; **(B)** OS in tumors overexpressing TMPRSS2.

**FIGURE 5:**
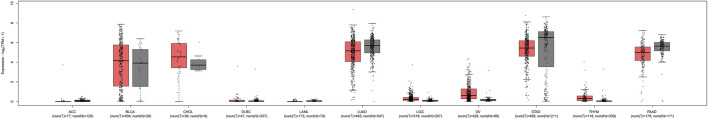
Mutations of TMPRSS2 in different tumors. **(A)** The mutation site of TMPRSS2 is displayed in the plot based on TCGA database from cBioPortal. **(B)** 3D structure of TMPRSS2 and the mutation site in TCGA. **(C)** The mutation site of TMPRSS2 is displayed in the plot based on the mixed pan-cancer cohort from cBioPortal. **(D)** 3D structure of TMPRSS2 and the mutation site in the mixed pan-cancer cohort.

**FIGURE 6:**
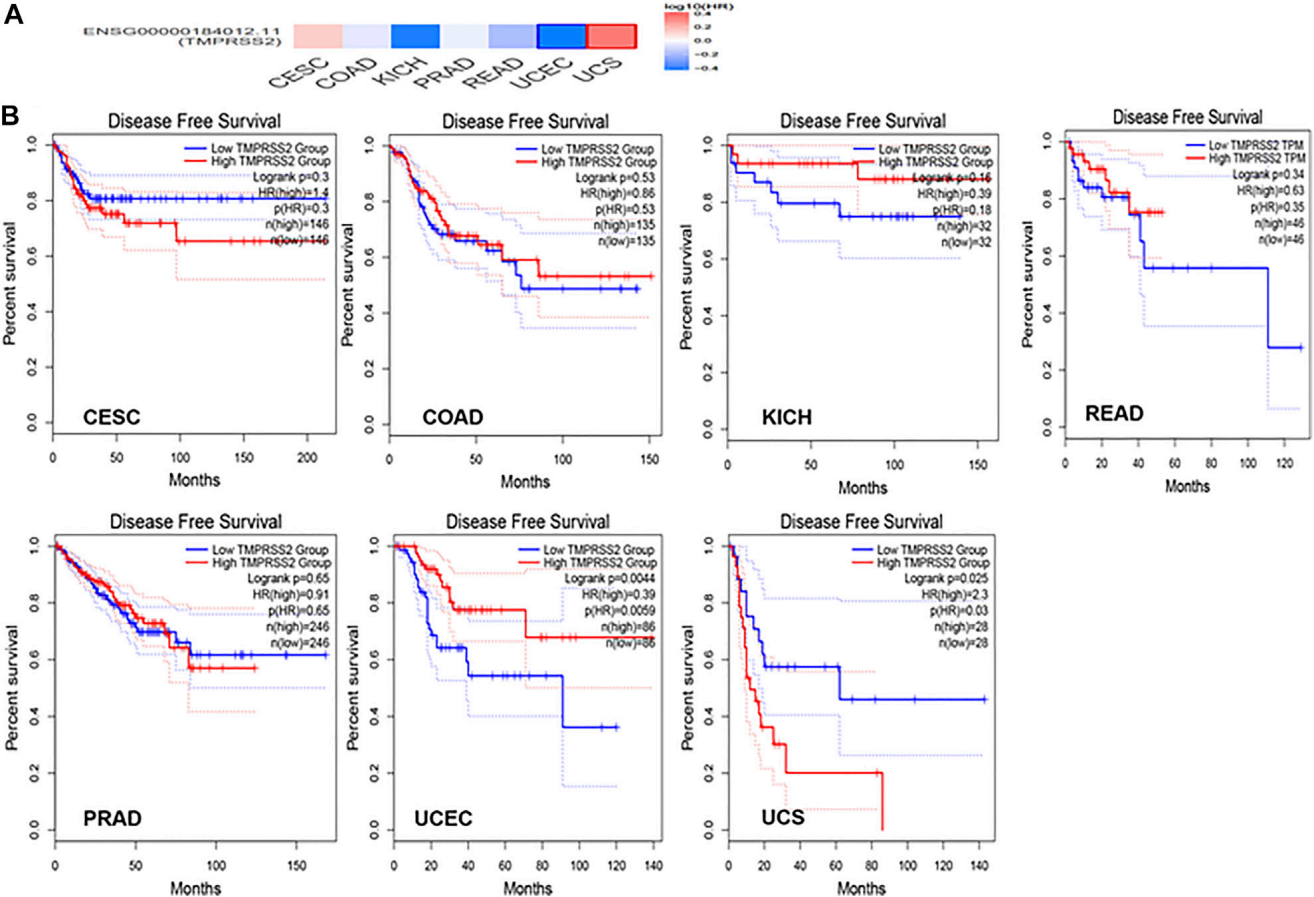
OS data of malignant tumors with low expression of TMPRSS2. **(A)** Survival picture of tumors with low expression of TMPRSS2; **(B)** OS in tumors with low expression of TMPRSS2.

**FIGURE 7:**
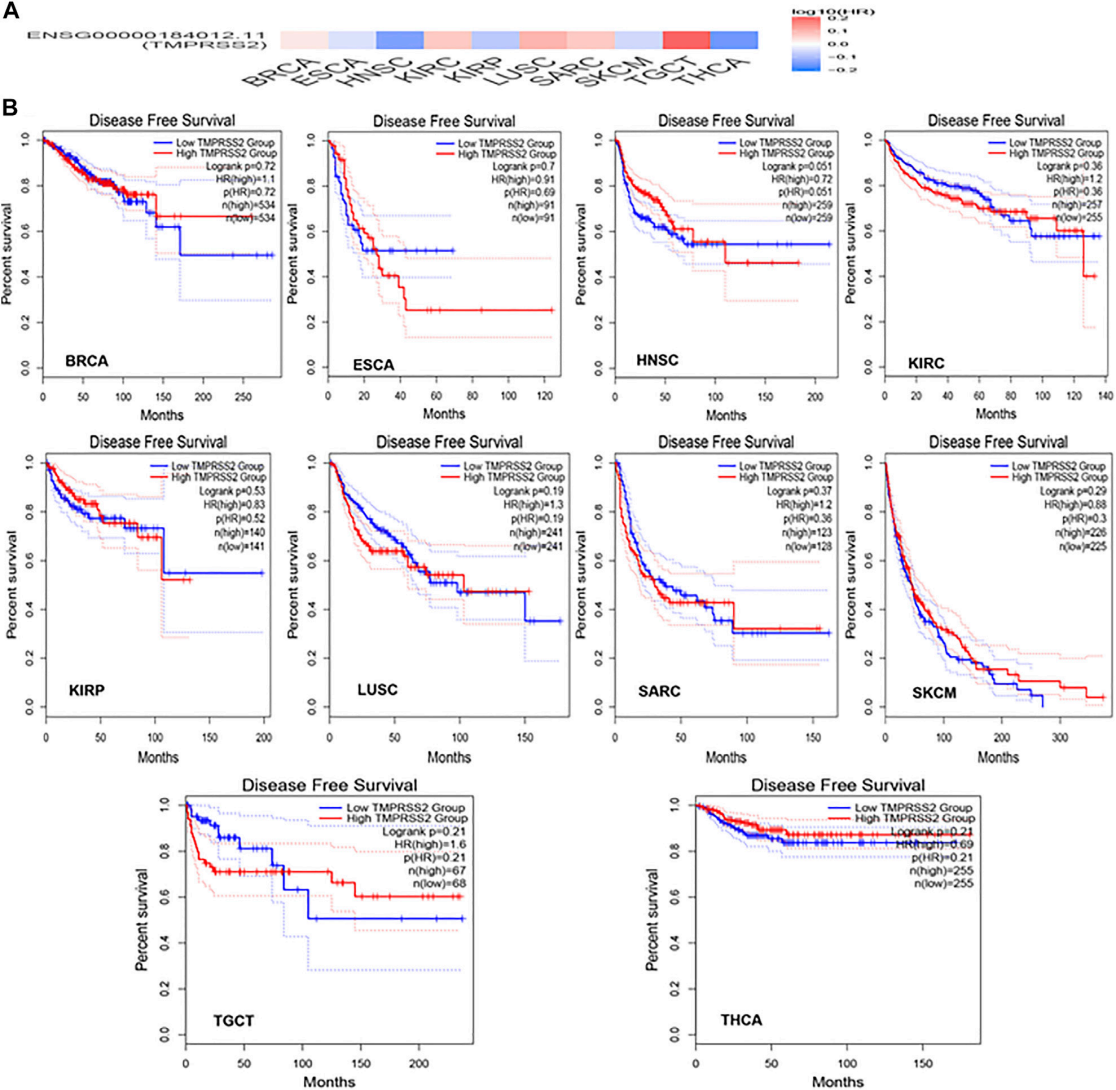
Correlation between genetic disorders and TMPRSS2 expression. **(A)** In most cases, there was no statistical correlation between DNA copy variation and RNA TMPRSS2 expression. **(B)** The mutation has nothing to do with RNA expression.

**FIGURE 8:**
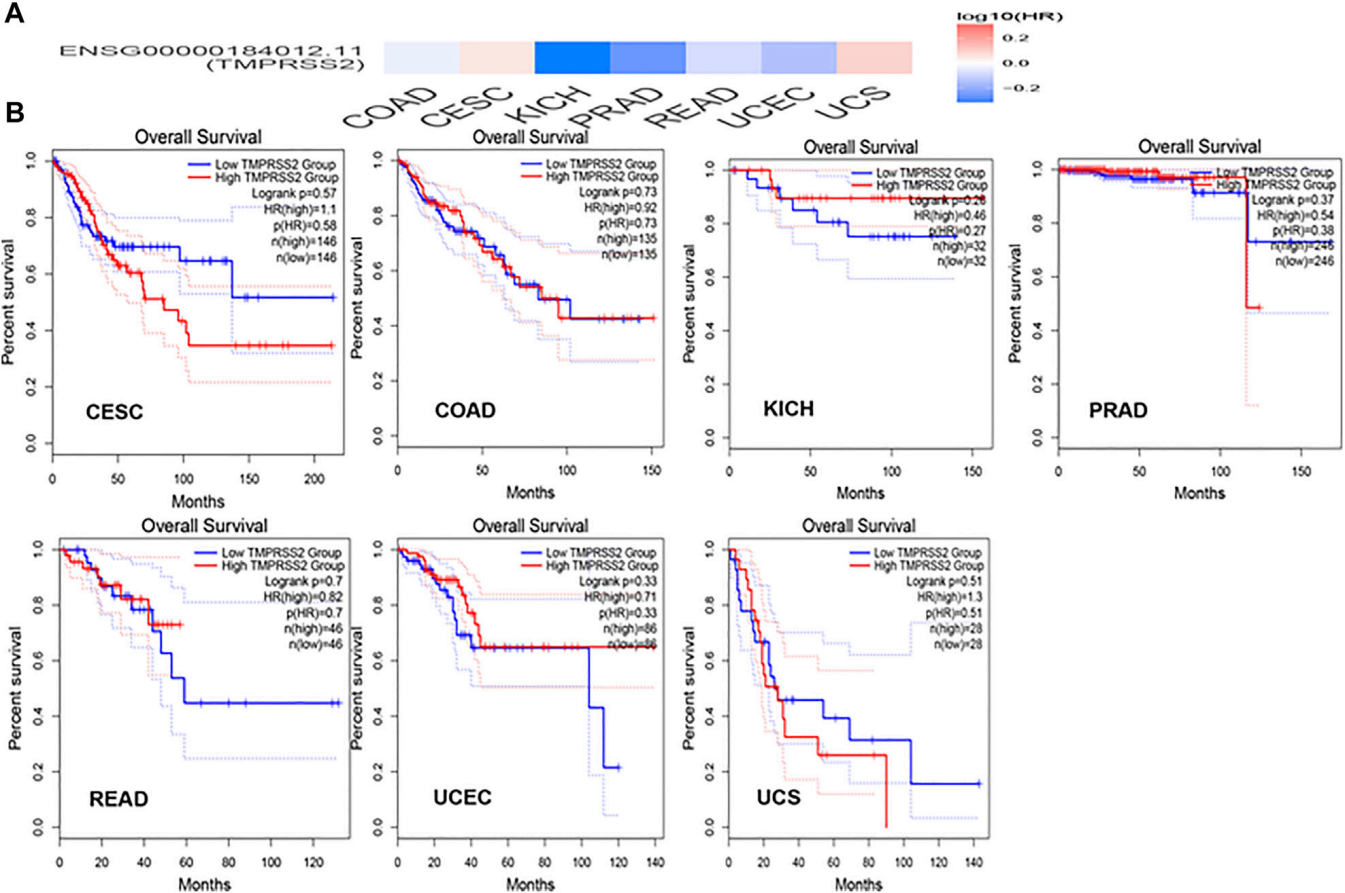
Expression level of TMPRSS2 in different pathological stages. Based on TCGA data, the expression level of TMPRSS2 was analyzed according to the main pathological stages (stage I, stage II, stage III, and stage IV) of KICH, ESCA, KIRC, TGCT, THCA, and KIRP.

**FIGURE 9:**
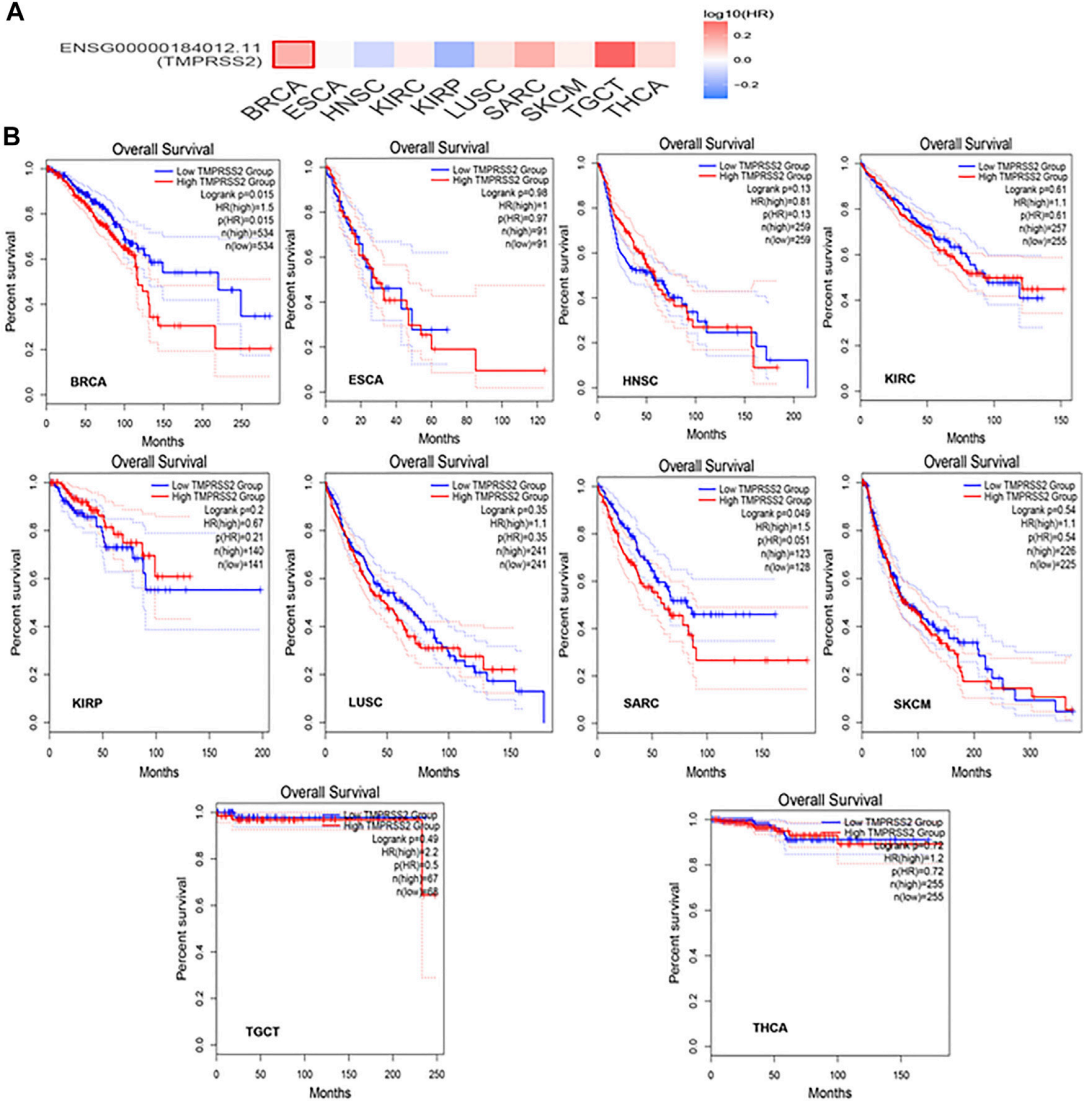
TMPRSS2 expression unchanged in some of the tumors. There was no difference in the expression of TMPRSS2 in some tumors and normal tissues, including adrenocortical carcinoma (ACC), bladder urothelial carcinoma (BLCA), cholangiocarcinoma (CHOL), lymphoid neoplasm diffuse large B-cell lymphoma (DLBC), acute myeloid leukemia (LAML), brain lower grade glioma (LGG), ovarian serous cystadenocarcinoma (OV), stomach adenocarcinoma (STAD), thymoma (THYM), pancreatic adenocarcinoma (PAAD), and even lung adenocarcinoma (LUAD).

GEPIA2 has been explored to dynamically analyze the differential expression of TMPRSS2 in normal and tumor tissues, including RNA sequencing expression data of more than 9,000 tumor samples and more than 8,000 normal samples from TCGA and GTEx. The data have proven that TMPRSS2 is overexpressed in colon adenocarcinoma (COAD), cervical squamous cell carcinoma and endometrial carcinoma (CESC), kidney chromophobe (KICH), prostate adenocarcinoma (PRAD), uterine corpus endometrial carcinoma (UCEC), uterine carcinosarcoma (UCS), and rectal adenocarcinoma (READ). However, the expression of TMPRSS2 in breast invasive cancer (BRCA), esophageal cancer (ESCA), head and neck squamous cell carcinoma (HNSC), kidney renal clear cell carcinoma (KIRC), kidney renal papillary cell carcinoma (KIRP), rectal adenocarcinoma (READ), sarcoma (SARC), skin cutaneous melanoma (SKCM), testicular germ cell tumor (TGCT), and thyroid cancer (THCA), including lung squamous cell carcinoma (LUSC), was downregulated (Figure 4). The mining data still revealed that there was no difference in the expression of TMPRSS2 in some tumors and normal tissues, including lung adenocarcinoma (LUAD) (Figure 5). The disease-free survival of UCES and UCS with high expression of TMPRSS2 was significantly different from that of the low-expression group (Figure 6 and Figure 7). The overall survival analysis found that BRCA with high expression of TMPRSS2 was associated with poor prognosis (Figure 8 and Figure 9).

### Methylation Level of TMPRSS2 in Pan-Cancer

**FIGURE 10:**
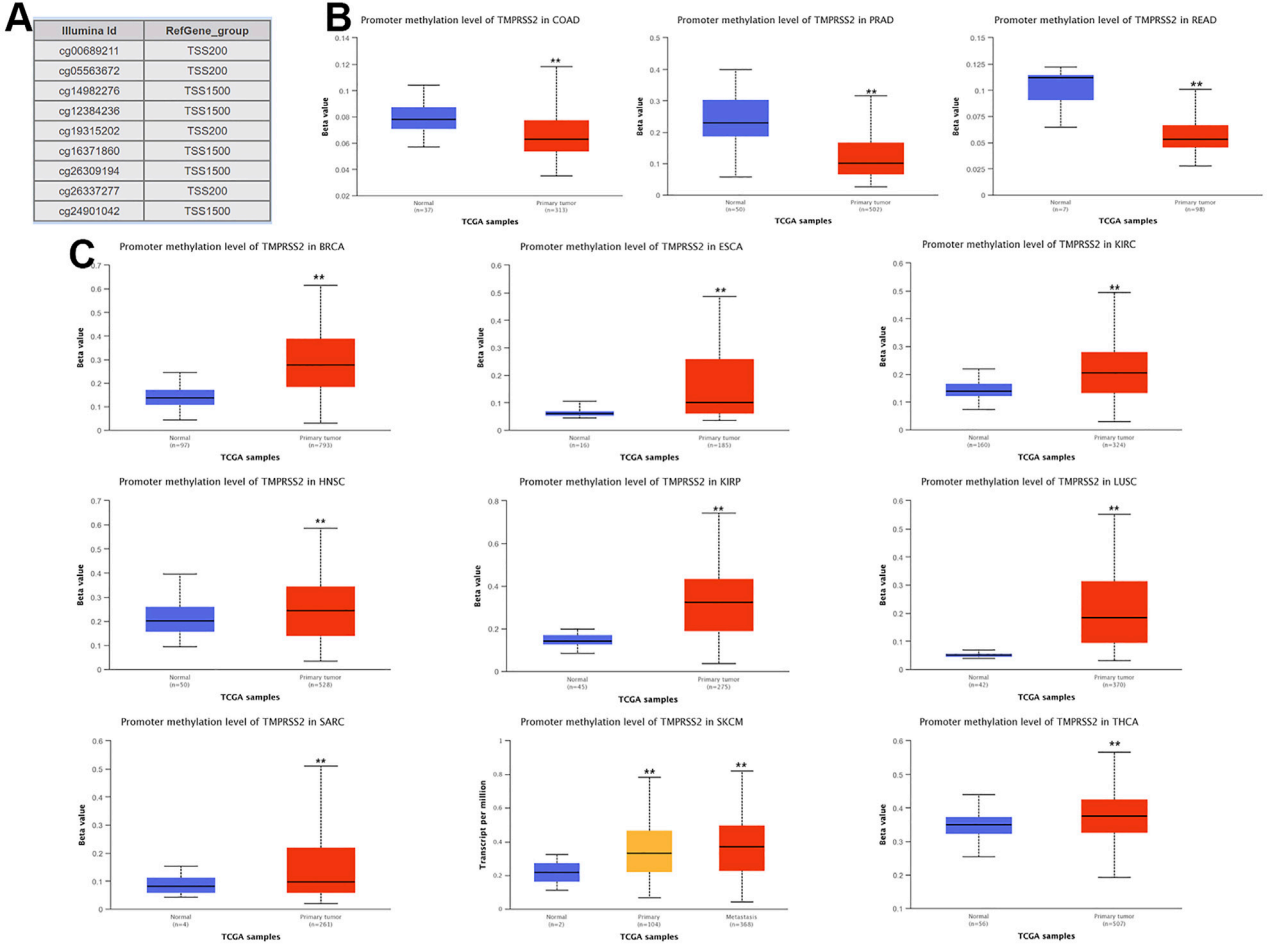
Disease-free survival (DFS) data in malignant tumors with TMPRSS2 overexpression. **(A)** Survival picture of tumors overexpressing TMPRSS2; **(B)** DFS in TMPRSS2-overexpressing tumors.

The potential relationship between TMPRSS2 DNA methylation and the pathogenesis of different tumors in the TCGA project was detected using the MEXPRESS method. In COAD, PRAD, and READ with high expression of TMPRSS2, the methylation level was lower than that of normal tissues, and the difference was statistically significant. In contrast, tumors with downregulated TMPRSS2 expression, such as BRCA, ESCA, HNSC, KIRC, KIRP, LUSC, SARC, SKCM, and THCA, displayed increased levels of DNA methylation, and the difference was statistically significant (Figure 10).

### The Relationship Between TMPRSS2 and Tumor Pathological Staging

**FIGURE 11:**
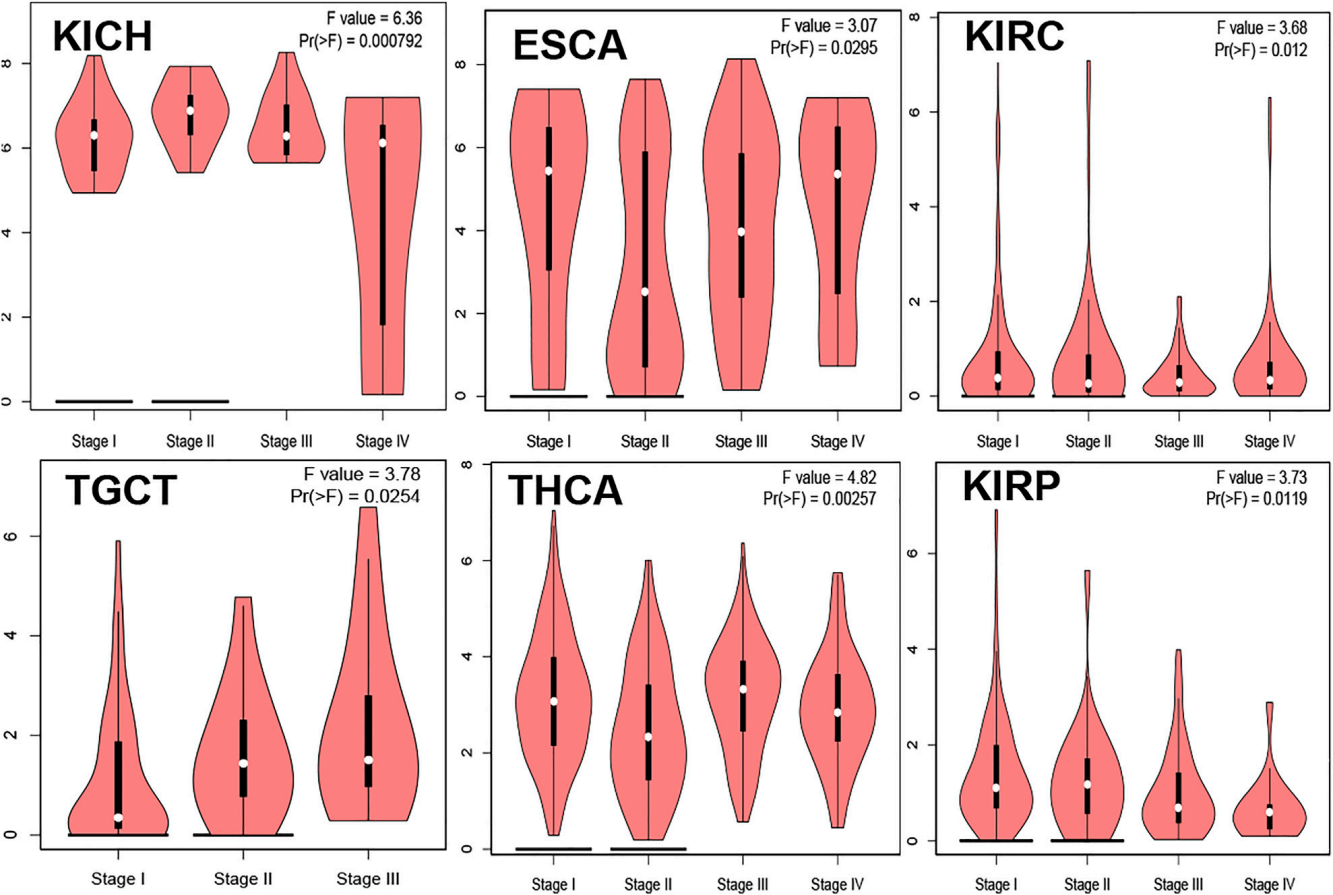
DFS data in malignant tumors with low TMPRSS2 expression. **(A)** Survival picture of tumors with low TMPRSS2 expression; **(B)** DFS in tumors with low TMPRSS2 expression.

The relationship between TMPRSS2 gene expression and KICH, ESCA, KIRC, TGCT, THCA, and KIRP pathological stages was analyzed and is presented in Figure 11, Pr (>F) ≤ 0.05. These results further indicated that the expression level of TMPRSS2 can function as a staging indicator for judging patients with KICH, ESCA, KIRC, TGCT, THCA, and KIRP.

### Correlation Analysis Between TMPRSS2 Expression and Tumor-Associated Fibroblast Immune Infiltration

**FIGURE 12:**
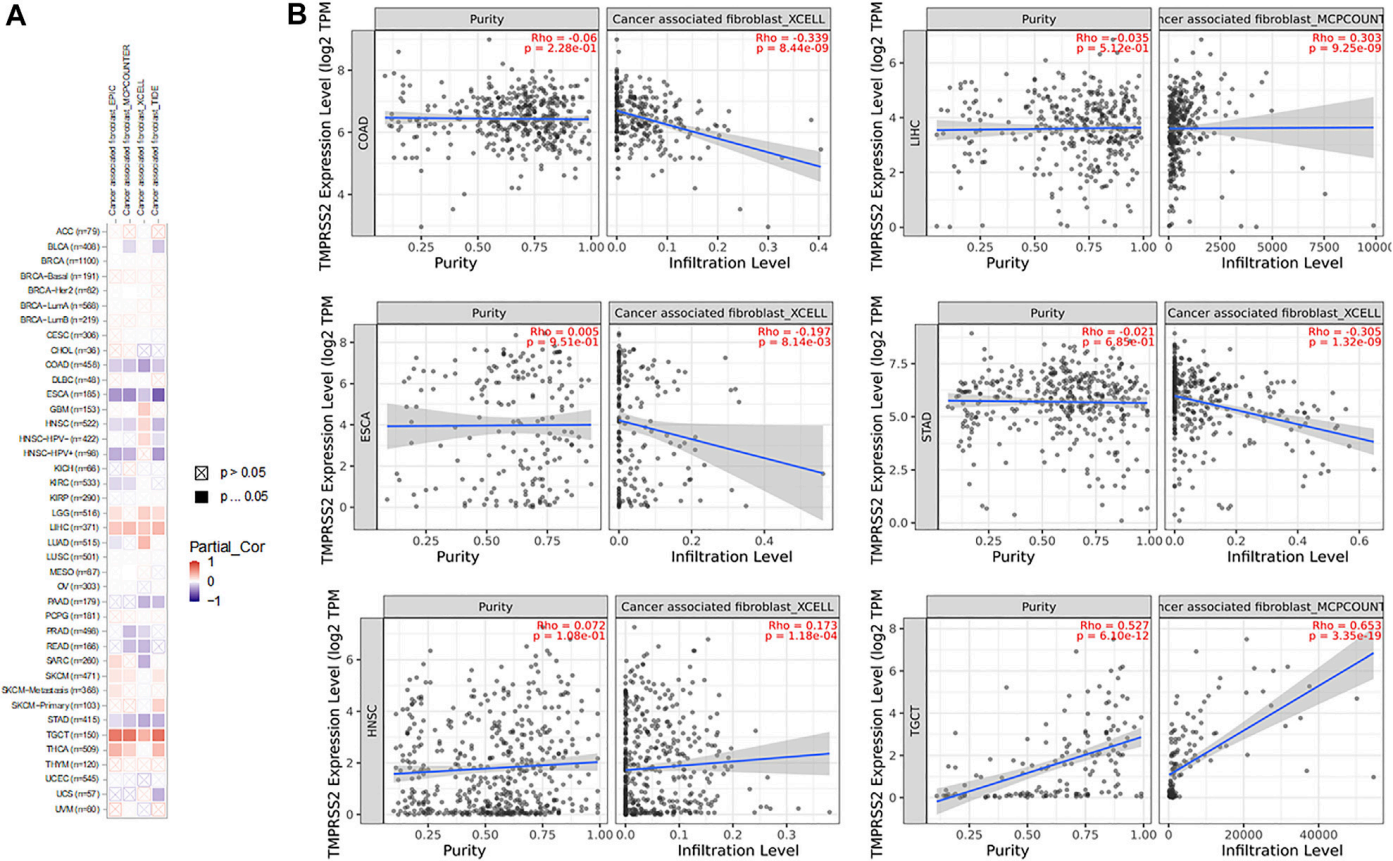
Correlation analysis of TMPRSS2 expression and immune infiltration of cancer-related fibroblasts. **(A)** Different algorithms were used to explore the potential correlation between the expression level of the TMPRSS2 gene and the infiltration level of cancer-related fibroblasts in all types of cancers in TCGA. **(B)** Enumerated XCELL infiltration of COPD, ESCA, HNSC, and STAD and MCPCOUNTER infiltration of LIHC and TGCT.

The TIMER database was explored to evaluate the relationship between TMPRSS2 expression and tumor-associated fibroblast immune infiltration in different tumor tissues. The TIMER, CIBERSORT, CIBERSORT-ABS, QUANTISEQ, XCELL, MCPCOUNTER, and EPIC algorithms were adopted to detect the potential relationship between different immune cell infiltration levels and TMPRSS2 gene expression in different cancer types. The findings illustrated that the expression of TMPRSS2 was closely related to the immune infiltration of tumor-associated fibroblasts in COPD, ESCA, HNSC, STAD, LIHC, and TGCT (Figure 12).

### Enrichment Analysis of TMPRSS2 Related Genes

**FIGURE 13:**
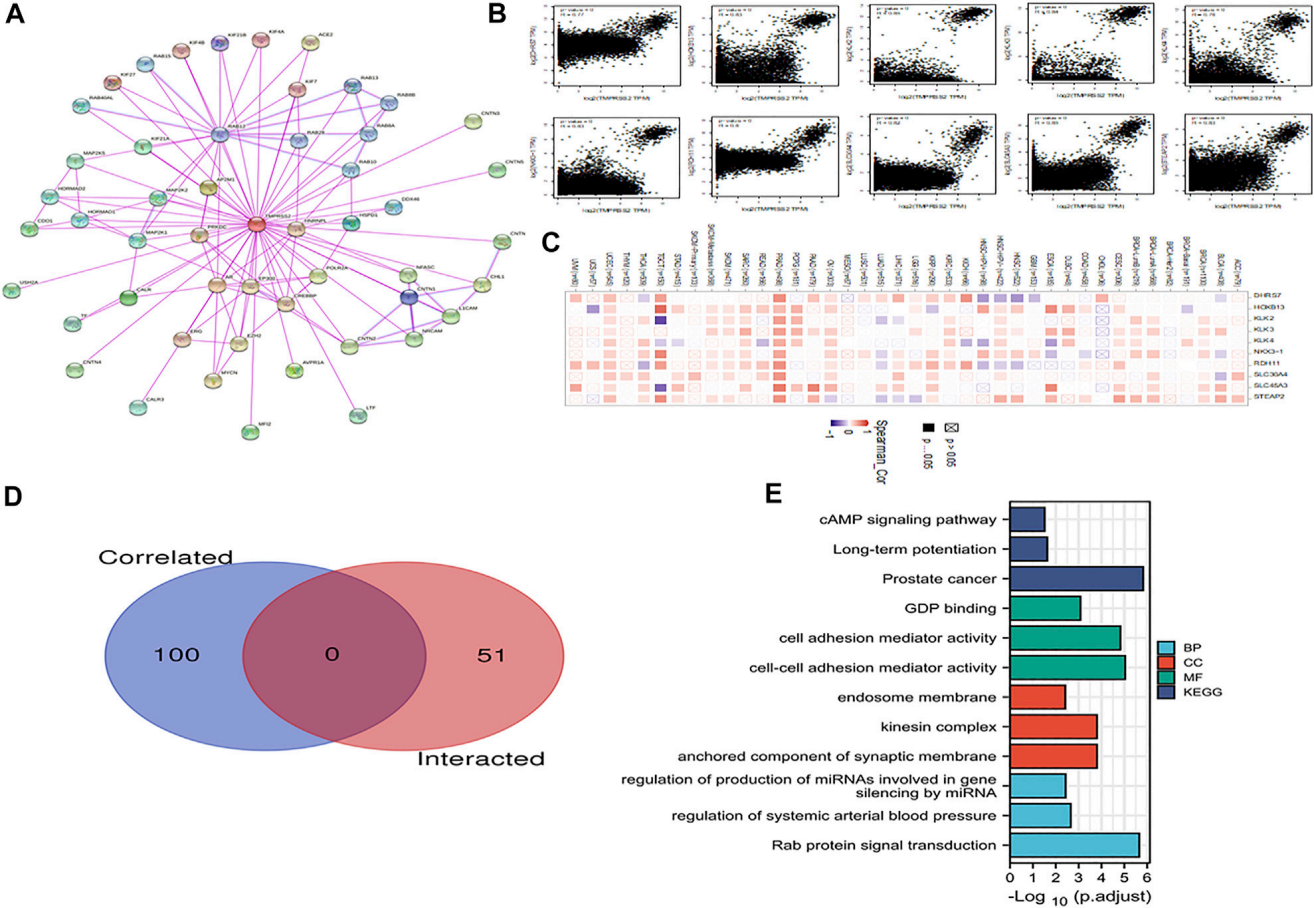
Enrichment analysis of TMPRSS2-related genes. **(A)** The STRING tool was utilized to obtain available TMPRSS2 binding proteins. **(B)** Using the GEPIA2 method, we also obtained the top 100 TMPRSS2-related genes in the TCGA project, including DHRS7, HOXB13, KLK2, KLK3, KLK4, NKX3-1, RDH11, SLC30A4, SLC45A3, and STEAP2. **(C)** The plot demonstrates the correlation data between TMPRSS2 and the related top 10 genes in various cancers. **(D)** Cross-analysis of TMPRSS2 binding genes and related genes was performed. **(E)** GO/KEGG pathway analysis based on TMPRSS2 binding genes and interacting genes.

To further investigate the molecular mechanism of the TMPRSS2 gene in tumorigenesis, the TMPRSS2 binding protein and TMPRSS2 expression–related genes for a series of pathway enrichment analyses were screened. Based on the STRING tool, the obtained binding proteins were supported by experimental evidence, and Figure 13A reveals the interaction network. The GEPIA2 tool was applied to obtain the top 100 genes related to TMPRSS2 expression. The corresponding heatmap data also indicated the correlation between TMPRSS2 and the top 10 genes in most detailed cancer types (Figure 13C). In addition, KEGG/GO enrichment analysis data further showed that most of these genes were related to endosome membrane, presynapse, Golgi vesicle transport, Ras protein signal transduction, axonogenesis, regulation of neuron projection development, prostate cancer, Rab protein signal transduction, and other related pathways or cell biology (Figures 13D,E).

## Discussion

Previous research evidence has shown that TMPRSS2 plays an important role in monitoring the infection process of severe acute respiratory syndrome (Khoury et al., 2020; Suarez-Farinas et al.) virus and Middle East respiratory syndrome (Lavin et al., 2017) coronavirus (Khan and Khan, 2021). Similarly, in a research report on SARS-CoV-2, TMPRSS2 and angiotensin converting enzyme 2 (ACE2) were coexpressed in human air and alveolar and blood air (Lukassen et al., 2020; Suarez-Farinas et al., 2021). ACE2 has been identified as a functional receptor for pathogenic SARS-CoV-2, which is related to the transport of the virus from the cell membrane to the cytoplasm (Barnes et al., 2020; Liu K. et al., 2021). TMPRSS2 promotes the binding of SARS-CoV-2 to ACE2 in the host cell by activating the S protein and assists the virus in entering the host cell (Kusmartseva et al., 2020). TMPRSS2 may also be the key to SARS-CoV-2 replication, and its expression greatly promotes the replication of the virus and the formation of syncytial virus–infected cells (Buchrieser et al., 2021; Kruger et al., 2021). The expression of TMPRSS2 greatly promoted the replication of the virus and the formation of syncytia (Iwata-Yoshikawa et al., 2019). Another study showed that in the presence of TMPRSS2, the number of SARS coronaviruses entering cells increased by 2.6 times, and the targeted elimination of TMPRSS2 could significantly reduce the number of SARS coronaviruses entering cells (Kawase et al., 2012). In addition, cancer has been identified as a personal factor of COVID-19, so the significance of TMPRSS2 expression in pan-cancers is more likely to be connected to the susceptibility of SARS-CoV-2 to tumor patients (Kuderer et al., 2020; Moris et al., 2020). Some researchers have suggested that although patients with underlying diseases are more susceptible to SARS-CoV-2 infection, patients suffering from head and neck cancer or lung cancer have reduced expression of TMPRSS2 in the body (Sacconi et al., 2020), which makes these patients less susceptible to SARS-CoV-2. Our research has genetically engineered the genetic and epigenetic variation of TMPRSS2 in humans for the first time.

TMRPSS2 expression was overexpressed in seven different types and downregulated in 10 different types (Figure 4). Since COVID-19 is mainly spread through the airway, we are particularly concerned about respiratory tumors. TMRPSS2 was significantly decreased in LUSC but remained unchanged in LUAD (Figure 5). Nine other types of tumors, including BRCA, ESCA, HNSC, KIRC, KIRP, SARC, SKCM, TGCT, and THCA, exhibited downregulation of TMPRSS2. The correlation between genetic diseases and TMRPSS2 expression was also confirmed in this research. In addition, there was no correlation between DNA/RNA mutations and TMRPSS2 expression (Figure 3). Our results implied that there are some differences in the frequency of TMPRSS2 variants in patients with different tumors. The existence of genetic variation does not always affect gene expression. Therefore, the upregulation of TMPRSS2 expression may not be caused by genetic mutations.

Nine probes in the TMRPSS2 promoter were used to detect the DNA methylation level of TMRPSS2 (Figure 10A). The findings verified that three tumors with high expression of TMPRSS2 manifested reduced levels of TMPRSS2 DNA methylation, including COAD, PRAD, and READ (Figure 10B). In other respects, among the nine tumors downregulated by TMPRSS2, BRCA, ESCA, HNSC, KIRP, SARC, SKCM, THCA, and even LUSC showed increased DNA methylation levels (Figure 10C). Since there is no DNA methylation dataset available for KICH normal controls, global DNA methylation levels of KICH cannot be compared. Thus, the DNA methylation levels of KICH at different tumor stages were compared, and the outcomes further found the enhancement of DNA methylation levels. In addition, the level of DNA methylation in other tumors with differential expression of TMPRSS2 remained unchanged, indicating that DNA methylation may not be the only cause of abnormal TMPRSS2 expression, such as histone modification and glycosylation. The high level of methylation in the promoter region can also silence the gene transcription process. Therefore, in some tumors, such as LUSC, the high level of methylation in the TMPRSS2 promoter may lead to a decrease in its transcriptional expression level.

The connection of clinical information with TMPRSS2 expression was monitored by the GEPIA2 database. The disease-free survival of UCES and UCS with high expression of TMPRSS2 was significantly different from that of the low-expression group (Figures 6, 7). The overall survival analysis found that BRCA with high expression of TMPRSS2 was related to poor prognosis. However, low expression of TMPRSS2 was irrelevant to the prognosis of tumor patients (Figures 8, 9). These data suggested that TMPRSS2 may be a double-edged sword in tumor patient prognosis. Therefore, other clinical features should also be fully considered. Second, more in-depth molecular experimental evidence is needed to determine whether the high expression of TMPRSS2 plays an important role in the occurrence of the aforementioned tumors or if it is just the result of normal tissues resisting tumor alterations.

As reported, TMPRSS2 has been shown to be an important regulator of tumorigenesis (Bao et al., 2020). TMPRSS2-ERG gene fusion occurs in approximately 50% of prostate cancer (PCa) cases, and the fusion product is a key driver of prostate cancer. Cell signaling to ablate the TMPRSS2-ERG oncoprotein may be beneficial in the treatment of PCa (Hong et al., 2020). Studies have shown that regardless of the size of the primary tumor, deletion of TMPRSS2 in tumor-bearing mice can significantly reduce metastasis (Kang et al., 2021). This is consistent with the emerging view that metastasis and primary tumor growth are controlled by different factors and suggests that TMPRSS2 may be essential for tumor metastasis behavior. By using a large number of single-cell RNA sequencing datasets, scientists systematically studied the expression of ACE2 and TMPRSS2 in human tumors and normal colorectal tissues and found that these two receptors are highly expressed in colorectal epithelial cells. They further found that patients with colorectal cancer and COVID-19 were more prone to lymphopenia with higher respiratory rates and high-sensitivity C-reactive protein levels than patients with COVID-19 alone (Liu C. et al., 2021). The expression of TMPRSS2 is downregulated in patients with head and neck cancer, which implies more resistance to SARS-CoV-2 infection (Sacconi et al., 2020).

In this research, the potential correlation between tumor-associated fibroblast immune infiltration in all TCGA tumors and TMPRSS2 was explored using the Timer 2.0 database. Information on TMPRSS2 binding components and TMPRSS2 expression–related genes in more than 30 tumors was obtained simultaneously by a series of enrichment analyses. The findings demonstrated that most of these genes were related to endosome membrane, presynapse, Golgi vesicle transport, Ras protein signal transduction, axonogenesis, regulation of neuron projection development, prostate cancer, Rab protein signal transduction, and other related pathways or cell biological functions.

In conclusion, the genetic changes, RNA expression, and DNA methylation of TMPRSS2 were analyzed in more than 30 tumors. These findings suggest the need for priority precautions for COAD, CESC, KICH, PRAD, UCEC, UCS, and READ during the COVID-19 pandemic. In addition, low DNA methylation of TMPRSS2 was also found in most of these tumors with high TMPRSS2 expression. In the end, our study planned the genetic and epigenetic variation of TMPRSS2 in human malignant tumors for the first time. However, the specific molecular mechanism of TMPRSS2 in the formation and development of tumors *in vivo* has not been clarified. Since our research is a bioinformatics test, it is necessary to conduct further functional and clinical verification.

## Data Availability

The original contributions presented in the study are included in the article/Supplementary Materials; further inquiries can be directed to the corresponding authors.
