# Supplementary material for: A Pan-Cancer In Silico Analysis of the COVID-19 Internalization Protease: Transmembrane Proteaseserine-2
Source: Front Genet. 2022 Feb 25;13:805880. doi: 10.3389/fgene.2022.805880 (PMC8913938; doi:10.3389/fgene.2022.805880)
Supplement: Supplementary file 1 [file DataSheet2.DOCX]

**Figure Legends**

**Fig. 1 Genetic variation of TMPRSS2 in tumors.**

**(A)** The TCGA pan-cancer panel was obtained from the cBioPortal dataset, which included 10953 patients/10967 samples in 32 studies. **(B)** The mixed pan-cancer panel was obtained from the cBioPortal dataset, which also included 46305 patients/48834 samples in 188 studies.

**Fig. 2 The RNA expression of TMPRSS2.**

**(A)** Exploration of the differential expression of TMPRSS2 through the GEPIA2 database (upper panel. Red, tumor samples; green, normal samples) and the UALCAN database (lower panel. Blue, tumor samples; Red, normal samples). **(B)** TMPRSS2 is overexpressed in colon adenocarcinoma (COAD), cervical squamous cell carcinoma and endocervical adenocarcinoma (CESC), kidney chromophobe (KICH), prostate adenocarcinoma (PRAD), uterine corpus endometrial carcinoma (UCEC), uterine carcinosarcoma (UCS) and rectum adenocarcinoma (READ). **(C)** TMPRSS2 is downregulated in breast invasive carcinoma (BRCA), esophageal carcinoma (ESCA), head and neck squamous cell carcinoma (HNSC), kidney renal clear cell carcinoma (KIRC), kidney renal papillary cell carcinoma (KIRP), lung squamous cell carcinoma (LUSC), sarcoma (SARC), skin cutaneous melanoma (SKCM), testicular germ cell tumors (TGCTs) and thyroid carcinoma (THCA).

**Fig. 3 Mutations of TMPRSS2 in different tumor.**

**(A)** The mutation site of TMPRSS2 is displayed in the plot based on TCGA database from cBioPortal. **(B)** 3D structure of TMPRSS2 and mutation site in TCGA. **(C)** The mutation site of TMPRSS2 is displayed in the plot based on the mixed pan-cancer cohort from cBioPortal. **(D)** 3D structure of TMPRSS2 and mutation site in the mixed pan-cancer cohort.

**Fig. 4 Methylation level of TMPRSS2 in pan-cancer.**

**(A)** TMPRSS2 promoter DNA methylation probe. **(B)** Three tumors with high expression of TMPRSS2 showed reduced levels of TMPRSS2 DNA methylation, including colon adenocarcinoma (COAD), prostate adenocarcinoma (PRAD) and rectal adenocarcinoma (READ), ** p <0.01. **(C)** Tumors with downregulated TMPRSS2 expression, breast invasive carcinoma (BRCA), esophageal carcinoma (ESCA), head and neck squamous cell carcinoma (HNSC), kidney renal clear cell carcinoma (KIRC), kidney renal papillary cell carcinoma (KIRP), lung squamous cell carcinoma (LUSC), sarcoma (SARC), skin cutaneous melanoma (SKCM) and thyroid carcinoma (THCA), exhibit increased levels of DNA methylation. ** p <0.01.

**Fig. 5 The correlation between genetic disorders and** **TMPRSS2 expression.**

**(A)** In most cases, there was no statistical correlation between DNA copy variation and RNA TMPRSS2 expression. **(B)** The mutation has nothing to do with RNA expression.

**Fig. 6 The expression level of TMPRSS2 in different pathological stages.**

Based on TCGA data, the expression level of TMPRSS2 was analyzed according to the main pathological stages (stage I, stage II, stage III and stage IV) of KICH, ESCA, KIRC, TGCT, THCA and KIRP.

**Fig. 7 TMPRSS2 expression unchanged in some of the tumors.**

There was no difference in the expression of TMPRSS2 in some tumors and normal tissues, including adrenocortical carcinoma (ACC), bladder urothelial carcinoma (BLCA), cholangiocarcinoma (CHOL), lymphoid neoplasm diffuse large B-cell lymphoma (DLBC), acute myeloid leukemia (LAML), brain lower grade glioma (LGG), ovarian serous cystadenocarcinoma (OV), stomach adenocarcinoma (STAD), thymoma (THYM), pancreatic adenocarcinoma (PAAD) and even lung adenocarcinoma (LUAD).

**Fig. 8 Disease-free survival (DFS) data in malignant tumors with TMPRSS2 overexpression.**

(A) Survival picture of tumors overexpressing TMPRSS2; (B) DFS in TMPRSS2-overexpressing tumors.

**Fig. 9 DFS data in malignant tumors with low TMPRSS2 expression.**

**(A)** Survival picture of tumors with low TMPRSS2 expression; **(B)** DFS in tumors with low TMPRSS2 expression.

**Fig. 10 Overall survival (OS) data for malignant tumors overexpressing TMPRSS2.**

**(A)** Survival picture of tumors overexpressing TMPRSS2; **(B)** OS in tumors overexpressing TMPRSS2.

**Fig. 11 OS data of malignant tumors with low expression of TMPRSS2.**

**(A)** Survival picture of tumors with low expression of TMPRSS2; **(B)** OS in tumors with low expression of TMPRSS2.

**Fig. 12 Correlation analysis of TMPRSS2 expression and immune infiltration of cancer-related fibroblasts.**

**(A)** Different algorithms were used to explore the potential correlation between the expression level of the TMPRSS2 gene and the infiltration level of cancer-related fibroblasts in all types of cancers in TCGA. **(B)** Enumerated XCELL infiltration of COPD, ESCA, HNSC and STAD and MCPCOUNTER infiltration of LIHC and TGCT.

**Fig. 13 Enrichment analysis of TMPRSS2 related genes.**

**(A)** The STRING tool was utilized to obtain available TMPRSS2 binding proteins. **(B)** Using the GEPIA2 method, we also obtained the top 100 TMPRSS2-related genes in the TCGA project, including DHRS7, HOXB13, KLK2, KLK3, KLK4, NKX3-1, RDH11, SLC30A4, SLC45A3 and STEAP2. **(C)** The plot demonstrates the correlation data between TMPRSS2 and the related top 10 genes in various cancers. **(D)** Cross-analysis of TMPRSS2 binding genes and related genes was performed. **(E)** GO/KEGG pathway analysis based on TMPRSS2 binding genes and interacting genes.
