# Supplementary material for: A Pan-Cancer In Silico Analysis of the COVID-19 Internalization Protease: Transmembrane Proteaseserine-2
Source: Front Genet. 2022 Feb 25;13:805880. doi: 10.3389/fgene.2022.805880 (PMC8913938; doi:10.3389/fgene.2022.805880)

**A pan-cancer *in silico* analysis of the COVID-19 internalization protease: transmembrane proteaseserine-2 (TMPRSS2)**

Yiming Meng^a^, Jing Sun^b^, Guirong Zhang^a^, Tao Yu^c,*^, Haozhe Piao^a,d^.

^a^Department of Central laboratory, Cancer hospital of China medical university, Liaoning Cancer Hospital & Institute. No. 44, Xiaoheyan road, Dadong district, Shenyang, 110042, China.

^b^Department of Biobank, Cancer hospital of China medical university, Liaoning Cancer Hospital & Institute. No. 44, Xiaoheyan road, Dadong district, Shenyang, 110042, China.

^c^Department of Medical Imaging, Cancer hospital of China medical university, Liaoning Cancer Hospital & Institute. No. 44, Xiaoheyan road, Dadong district, Shenyang, 110042, China.

^d^Department of Neurosurgery, Cancer hospital of China medical university, Liaoning Cancer Hospital & Institute. No. 44, Xiaoheyan road, Dadong district, Shenyang, 110042, China.

^*^Corresponding author at: Department of Medical Imaging, Cancer hospital of China medical university, Liaoning province Cancer Hospital. No. 44, Xiaoheyan road, Dadong district Shenyang, 110042, China; Department of Neurosurgery, Cancer hospital of China medical university, Liaoning province Cancer Hospital. No. 44, Xiaoheyan road, Dadong district Shenyang, 110042, China

Tel: 86-180-413-94586 & 86-131-667-65053. Fax: 86-24-31916472.

Email: [taoyulnszlyy@sina.com](mailto:taoyulnszlyy@sina.com) & haozhepiaolnzlyy@sina.com

The first step for SARS-CoV-2 to enter the host is the process in which the virus particles controlled by the viral envelope glycoprotein bind to the host surface receptor and fuse with the cell membrane. Studies have shown that activation of virus by host protease is a prerequisite for membrane fusion. The choice of protease determines the location of cell membrane fusion, and the combination of virus and ACE2 receptor can trigger the uptake of virus particles by the host cell's intracellular body, so that TMPRSS2 could cut S protein in assisting virus infection. TMPRSS2 is mainly expressed in prostate epithelial cells, and a small amount is expressed in colon, lung, liver, kidney, pancreas and other epithelial cells, and is regulated by androgens and their receptors. TMPRSS2 can be self-activated, and the activated serine protease region falls off the cell membrane to mediate the protease cascade reaction. This study found that TMPRSS2 is widely expressed in different tumors. It was also found that in lung adenocarcinoma and lung squamous cell carcinoma, the transcription level of TMPRSS2 was decreased compared with normal lung tissue. In order to explore the reasons for the decreased expression of TMPRSS2, analysis of its methylation level in lung cancer found that the promoter methylation level of TMPRSS2 showed a significant increase. The high level of methylation in the promoter region can silence the transcription process of genes. Therefore, the high level of methylation of the TMPRSS2 promoter in lung adenocarcinoma and lung squamous cell carcinoma may cause its transcriptional expression level to decrease.


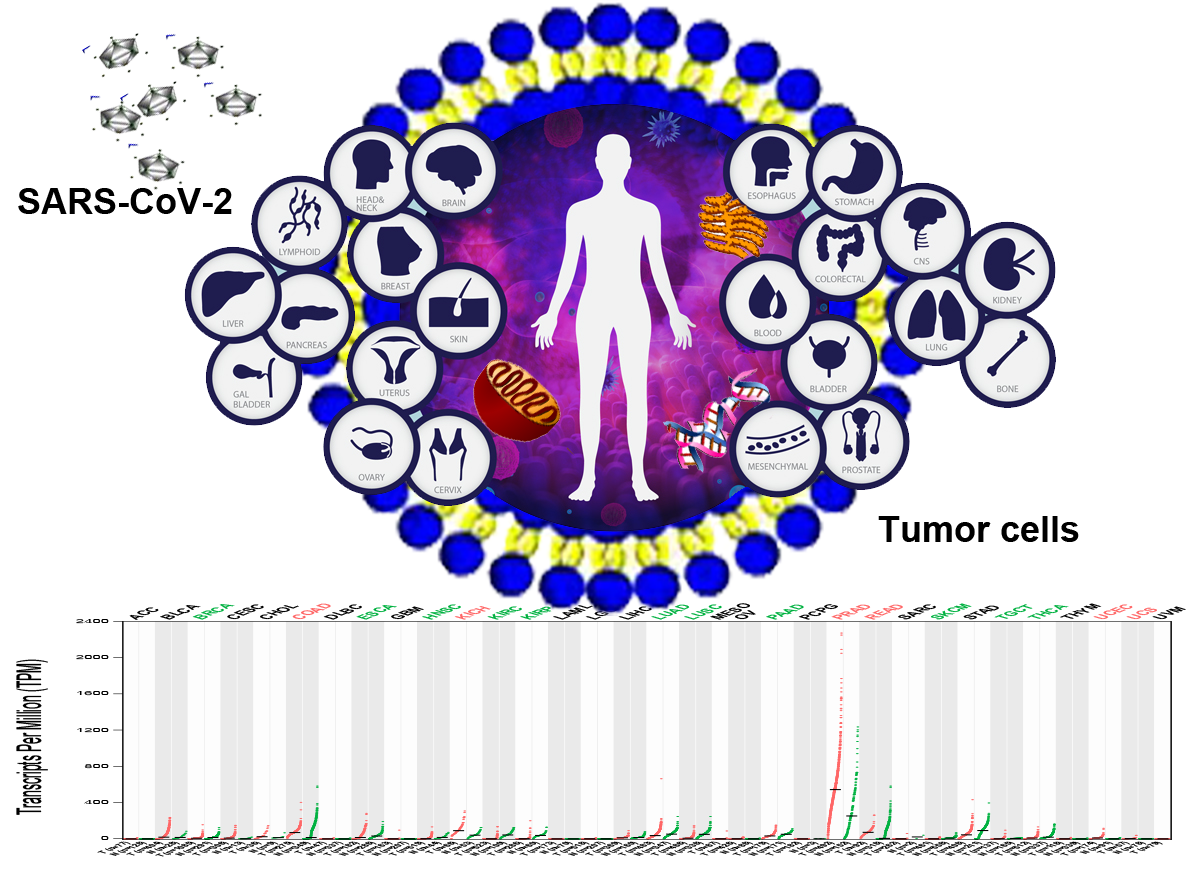

Supplement: Supplementary file 2 [file DataSheet1.DOCX]
